# Accelerated Repair and Reduced Mutagenicity of DNA Damage Induced by Cigarette Smoke in Human Bronchial Cells Transfected with *E.coli* Formamidopyrimidine DNA Glycosylase

**DOI:** 10.1371/journal.pone.0087984

**Published:** 2014-01-31

**Authors:** Mara Foresta, Alberto Izzotti, Sebastiano La Maestra, Rosanna Micale, Alessandro Poggi, Donatella Vecchio, Guido Frosina

**Affiliations:** 1 Department of Health Sciences, University of Genova, Genova, Italy; 2 Molecular Oncology and Angiogenesis Unit, IRCCS Azienda Ospedaliera Universitaria San Martino - IST Istituto Nazionale per la Ricerca sul Cancro, Genova, Italy; 3 Mutagenesis Unit, IRCCS Azienda Ospedaliera Universitaria San Martino - IST Istituto Nazionale per la Ricerca sul Cancro, Genova, Italy; University of Pittsburgh, United States of America

## Abstract

Cigarette smoke (CS) is associated to a number of pathologies including lung cancer. Its mutagenic and carcinogenic effects are partially linked to the presence of reactive oxygen species and polycyclic aromatic hydrocarbons (PAH) inducing DNA damage. The bacterial DNA repair enzyme formamidopyrimidine DNA glycosylase (FPG) repairs both oxidized bases and different types of bulky DNA adducts. We investigated *in vitro* whether FPG expression may enhance DNA repair of CS-damaged DNA and counteract the mutagenic effects of CS in human lung cells. NCI-H727 non small cell lung carcinoma cells were transfected with a plasmid vector expressing FPG fused to the Enhanced Green Fluorescent Protein (EGFP). Cells expressing the fusion protein EGFP-FPG displayed accelerated repair of adducts and DNA breaks induced by CS condensate. The mutant frequencies induced by low concentrations of CS condensate to the *Na^+^K^+^-ATPase* locus (oua^r^) were significantly reduced in cells expressing EGFP-FPG. Hence, expression of the bacterial DNA repair protein FPG stably protects human lung cells from the mutagenic effects of CS by improving cells’ capacity to repair damaged DNA.

## Introduction

Cigarette smoke (CS) is a self-inflicted damaging agent associated with high risk of developing chronic-degenerative diseases including cancer, obstructive pulmonary disease, and cardiovascular diseases. An important role of DNA damage has been recognized in the pathogenesis of these diseases [1]. Condensate from cigarette smoke (CSC) is mutagenic and genotoxic in nearly all systems in which it has been tested. In vivo, CS produces mutagenic urine and is a human somatic-cell mutagen producing hypoxanthine phosphoribosyltransferase mutations, sister chromatid exchanges, microsatellite instability and DNA damage in a variety of tissues. Smoking-associated genotoxic effects have been found in most of the twelve organ sites at which smoking causes cancer in humans [2] and lung tumors of smokers contain a high frequency and unique spectra of *TP53* and *KRAS* mutations [3].

Major mutagenic components of CS are polycyclic aromatic hydrocarbons (PAH), aromatic amines and N-nitrosamines that produce adducts on DNA. Those adducts are repaired in human cells by a network of DNA repair pathways including the nucleotide excision repair (NER) and the DNA base excision repair (BER) pathways. Recent research approaches aimed to determine whether induction of the BER enzyme OGG1 in lung cells by 7,8-dihydroxyflavone may protect these cells from the DNA damaging effects of smoking [4]. The 30.2 kDa formamidopyrimidine DNA glycosylase (FPG) is a BER protein that directly removes the damaged base and subsequently cleaves the resulting AP site by its associated β,δ AP lyase activity [5], [6]. Although some bulky adducts (e.g. N^7^-benzyl-FapydG) may be bound by FPG in an unproductive mode (i.e. with the FPG protein stalled at the damaged site - [6]), some others can be accommodated in the versatile catalytic site of FPG with removal of the damaged base [7]–[9]. We and others have demonstrated that heterologous expression of FPG in human cells stably protects from accumulation of various types of mutagenic DNA damages [(gene prophylaxis) reviewed in [10]]. We report here that expression of FPG in human lung cells stably enhances DNA repair of damage induced by cigarette smoke condensate (CSC) and reduces its mutagenicity.

## Materials and Methods

### Cell Lines

NCI-H727 (re-named H727 throughout) cells derive from a non small cell lung carcinoma of a 65 years old Caucasian woman. This cell line was chosen for the experiments reported here for being a well-differentiated bronchial carcinoid cell line with elevated proliferation rate and transfection efficiency as compared to normal (untransformed) human lung fibroblasts. It was purchased from European Collection of Cell Cultures (ECACC) via Interlab Cell Line Collection (ICLC) at IRCCS AOU San Martino – IST, Genova, Italy and cultured in RPMI 1640+10% FBS +2 mM L-Glutamine. H727 cells express easily detectable levels of *TP53* mRNA.

The H1 clone was derived from H727 cells by transfection with the pEGFP-C1 vector (vector only) [11]. H1 cells express the EGFP protein and were used in this study as a control cell line. To obtain clones expressing the fusion protein EGFP–FPG, H727 cells were transfected with the pEGFP-C1-FPG vector [11]. HF12 and HF45 cells indicate 2 independent clones of H727 cells transfected with the pEGFP-C1-FPG vector and expressing the fusion protein EGFP–FPG. H1, HF12 and HF45 cells were grown in the same medium of H727 cells supplemented with 800 µg/ml geneticin (G418).

### EGFP-FPG Vector and Cell Transfection

**Figure 1:**
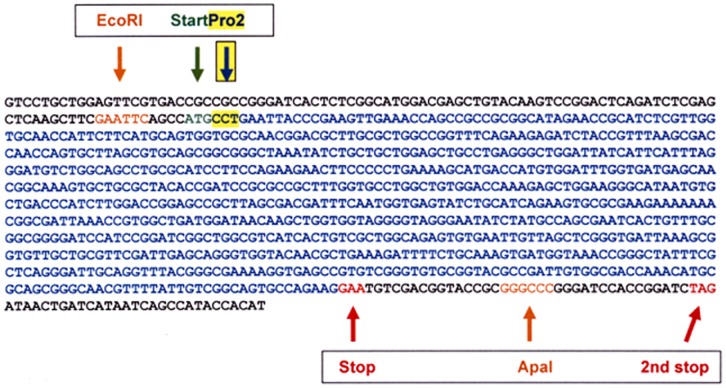
Sequence of FPG cloned in the pEGFP-C1 expression vector.

The construction of the pEGFP-FPG mammalian expression vector has been previously described [11]. Briefly, the *E.coli* FPG cDNA was cloned out from a pSF91.1 vector and inserted into the pEGFP-C1 mammalian expression vector (BD Biosciences, Franklin Lakes, NJ) using the cloning sites EcoRI–ApaI. The FPG gene cloned into the multiple cloning site is expressed as fusion to the C-terminus of the fluorescent protein EGFP (excitation maximum = 488 nm; emission maximum = 507 nm). The sequence of the cloned FPG gene with prolin (Pro) 2 acting as a nucleophile in the glycosylase/AP lyase reaction [12], [13], is shown in Figure 1. Vector-only plasmids were used as control.

Transfection of vectors was performed with Effectene Reagent (Qiagen, Hilden, Germany) according to manufacturer instructions with minor modifications. Briefly, 2×10^5^ H727 cells were seeded in 60-mm dishes; after 24 h, a mix of 1 µg linearized vector DNA, 8 µl Enhancer, and 25 µl Effectene reagent was added to the culture medium (1∶25 vector DNA/Effectene ratio). Cells were allowed to uptake vectors for 24 h and then washed and plated in 100-mm dishes. 100 µg/ml of Geneticin (G418) was added immediately after cell adhesion. G418 concentration was gradually raised to 800 µg/ml during the following 72 h. G418-resistant clones were screened under an inverted IX51 fluorescence microscope equipped with an XC30 digital colour camera and cellSens Entry software (Olympus Biosystems, Planegg, Germany). Nineteeen fluorescent HF (*H*727 with *F*PG insert) clones were isolated and two of them (HF12 and HF45) were selected for further studies on the basis of their fluorescence and proliferation capacity. Confocal microscopy of the cells was carried out at 600X magnification by an IX81 microscope equipped with FV500 imaging system (Olympus Biosystems, Planegg, Germany). Image analysis was carried out by FluoView 2.1 software.

### EGFP-FPG Expression

Cell fluorescence was analyzed under the inverted microscope and images were taken under both transmitted light and fluorescence. Immunodetection was carried out as described [14]. Briefly, 30 micrograms of extract protein prepared according to Biade and coworkers [15] were resolved onto each of two 12% SDS–PAGE minigel and electroblotted onto Hybond-C Extra nitrocellulose membrane (Amersham, Milano, Italy). Membranes were stained with Ponceau red to check the blotting efficiency, washed with distilled water, and blocked at room temperature in PBS containing 0.1% Tween +2% nonfat dry milk. One membrane was incubated with anti-FPG rabbit polyclonal antibody (1∶3500, overnight at 4°C, R&D Systems, Minneapolis, MN) and subsequently with peroxidase-conjugated goat anti-rabbit IgG (whole molecule; Sigma, St. Louis, MO) at a dilution of 1∶10000 for 1 h at room temperature. The second membrane was incubated with anti-EGFP rabbit polyclonal antibody (0.75 µg/ml; over night at 4°C, BD Biosciences, Franklin Lakes, NJ) and subsequently with peroxidase-conjugated goat anti-rabbit IgG (whole molecule; Sigma, St. Louis, MO) at a dilution of 1∶10000 for 1 h at room temperature. Immune complexes were visualized by the enhanced chemiluminescence (ECLplus) system (Amersham Biosciences, Milano, Italy).

### Clonogenicity

Cells were gently treated with 0.05% trypsin/0.2% EDTA to obtain single-cell suspensions and adjusted to clonal density 4–18 cells/cm^2^ and seeded in triplicates. The cultures were incubated for 15 days at 37°C, 5% CO_2_ and the medium was refreshed every 3–4 days. Every day the cells were controlled microscopically to ensure the single-cell origin of the clones. At the end of the incubation period, the cells were washed with PBS (pH 7.2) and stained with Giemsa solution following routine histological technique. Clusters of cells were considered colonies when they were visible macroscopically and contained >50 cells. Cloning efficiency (CE) was determined from the formula CE (%) = (number of colonies/number of cells seeded)×100.

### Population Doubling Time

The population doubling times (PDT) were determined according to Glaab and Tindall [16]. Briefly, 10^5^ cells were seeded in 25 cm^2^ flasks and the total number of cells was counted at the indicated hours of culture. After determination of the CE, the population doublings (PD) were determined using the equation: PD = [ln (total number of cells) - ln (number of cells plated × CE)]/ln2.

### Preparation of CSC

CSC was prepared as previously described [17]. Research cigarettes without filters (Kentucky Reference Cigarette 2R1; Tobacco Research Institute, University of Kentucky; Lexington, KY) were used. These cigarettes contain the four major tobacco types, including Flue-cured, Burley, Oriental and Maryland, in proportions to be representative of US cigarettes. The mainstream smoke of each 2R1 cigarette contains 38.8 mg of total particulate matter, 32.9 mg of tar (US Federal Trade Commission), 2.2 mg of nicotine, 3.7 mg of water, 22.2 mg of carbon monoxide, and 0.3 mg of nitrogen oxide. The mainstream smoke of two 2R1 cigarettes was aspirated by means of a pump (model TD-3LSA; Brailsford; Rye, NY) and collected onto a 25-mm filter (EMFab filter; Pall; East Hills, NY), which is composed of borosilicate glass microfibers reinforced with woven glass cloth and bonded with polytetrafluoroethylene. These filters are commonly used in critical aerosol sampling tests, such as diesel exhausts. The smoke was diluted in 50 ml of culture medium and then filtered through a 0.22-µm syringe filter (Millipore Corp; Bedford, MA) to remove bacteria and large particles. This solution was considered to be 100% CSC (0.04 cigarettes per milliliter).

### CSC-induced DNA Adducts

Evaluation of DNA adducts was performed by ^32^P postlabelling, following butanol enrichment. ^32^P-post-labelling, monodirectional thin layer chromatography and electronic autoradiography were carried out as previously described [18]. Briefly, DNA (10 µg) was depolymerized to 3′-monophosphate nucleotides by incubation with micrococcal nuclease (0.04 U/µg DNA) and spleen phosphodiesterase (1 mU/µg DNA) at 37°C for 3.5 h. DNA was incubated with T4 phage polynucleotide kinase (8 U) in the presence of ATgamma-^32^P (64 µCi, specific activity-750 Ci/mmol) (ICN, Irvine, CA) for 40 min at 37°C. ^32^P -labelled bulky DNA adducts were purified from the reaction mixture by monodirectional thin layer chromatography in 8.5 M Urea and identified by electronic autoradiography (Instant Imager, Packard, Meriden, CT). Benzo[a]pyrene-diolepxide-N2-dG (BPDE) and DNA-free samples were used as positive and negative control, respectively. BPDE-DNA adduct standard was used to control the efficiency of ^32^P postlabelling procedure in identifying DNA adducts and was not aimed at evaluating FPG activity.

### CSC-induced DNA Breaks

We used the SCGE assay under alkaline conditions as described [19]. Briefly, appropriate numbers of cells were treated in tissue culture flasks at 37°C with CSC for 72 hours. Cells were washed twice, resuspended in low melting point agarose and the mixture was placed over slides. After the solidification of the agarose, the slides were immersed in a lysing solution (2.5 M NaCl, 100 mM Na_2_-EDTA, 10 mM Tris-HCl pH10, fresh 10% DMSO, 1% Triton X-100) for 1 h to lyse the cells and to permit DNA unfolding. The slides were then removed from the lysing solution, washed with 10 mM Tris-HCl pH 10 and placed on a horizontal gel electrophoresis unit. The unit was filled with fresh electrophoretic buffer (1 mM Na_2_-EDTA and 300 mM NaOH) to a level 0.25 cm above the slides. The slides were allowed to set in this high-pH buffer for 20 min to allow unwinding of DNA before electrophoresis. Electrophoresis was conducted for the next 30 min at 25 V. Thereafter the slides were washed gently by placing them horizontally and flooding them slowly with 0.4 M Tris, pH 7.5. After 5 min, the slides were stained with ethidium bromide (20 µg/ml). Observations were made using a fluorescent microscope (Leica DMI 4000B, Leica, Wetzlar, Germany) equipped with a digital camera (Leica DFC 310FX), using an excitation filter offset at 515 nm and a barrier filter at 590 nm. Digital photomicrographs of single cells were taken at 400X magnification. The extent of DNA damage was evaluated as the distance of DNA migration from the body of the nuclear core, tail length (TL). TL was measured in at least 100 randomly selected cells in each treatment group at the indicated repair times by the Comet Assay Software Programme (http://casplab.com/). DNA breaks induced by ionizing radiation (IR) in complete medium were analyzed 20 minutes after treatment with 3 and 6 Gy delivered by a CIS Bio International IBL437C irradiator at a fluence of 0.13 Gy/sec.

### CSC-induced Toxicity

10^4^ cells were seeded in 96-well plates. The following day, cells were incubated in complete medium with the indicated doses of CSC or an equivalent volume of solvent [dimethylsulphoxide (DMSO) 1.9% v/v] at 37°C for 72 hours with no medium change. Cells were then washed twice with PBS and survival was evaluated by the MTT viability test [3-(4,5-dimethylthiazol-2-yl)-diphenyltetrazolium bromide]-based as per manufacturer’s instructions (Sigma, St. Louis, MO, USA).

### CSC-induced Mutagenicity

Cells (3×10^6^) were seeded in 75 cm^2^ flasks. The following day, the cells were exposed to the indicated concentrations of CSC in complete medium for 72 h and then washed. Cells were cultured for an expression period of 9 days, after which they were plated in 100 mm petri dishes (2×10^6^ cells/dish) and exposed to 1 µM ouabain. The colonies were fixed and counted after 30 days. Seeding of 500 cells/dish accompanied each selection for determination of viable cells.

### Cell Cycle Distribution after CSC Treatment

1.2–1.6×10^5^ cells were plated in 35 mm petri dishes and exposed 18 hours later to the indicated concentrations of CSC in complete medium for 72 h. Cells were then harvested by PBS-EDTA treatment, washed with PBS, fixed in 70% ethanol/water, permeabilized with 0.0015% NP40, washed and stained with 50 µg/ml propidium iodide (PI) solution containing 100 units/ml RNase type A, 10 mM EDTA. Samples were run on CyAn ADP cytofluorimeter (Beckman-Coulter, Brea,CA, USA) and analyzed by the ModFit 3.2 computer program (Verity Software House, Topsham, Maine, USA).

### Statistics

The two-tailed ‘Student’s’ t test was used. Statistical significance (p<0.05) was indicated with one star. Statistical analysis was performed using the GraphPad Prism 5.01 software for Windows.

## Results

The studies described here have been performed in transformed, immortal adenocarcinoma cells in order to achieve permanent expression of EGFP-FPG and investigate its effects on CSC mutagenicity at a target locus [*Na^+^/K^+^ ATPase* (oua^r^)]. Although untransformed lung cells may represent in this respect a more interesting material being more similar to normal lung tissues, the studies reported here would not have been feasible with these mortal cells as they only allow a limited number of cell divisions (see e.g. http://www.lonza.com/products-services/bio-research/primary-and-stem-cells/human-cells-and-media/fibroblasts-and-media/nhlf-normal-human-lung-fibroblasts.aspx). It should be yet emphasized that the data generated in an adenocarcinoma cell line may not be indicative of what happens in normal, non-cancer cells.

### FPG Expression

**Figure 2:**
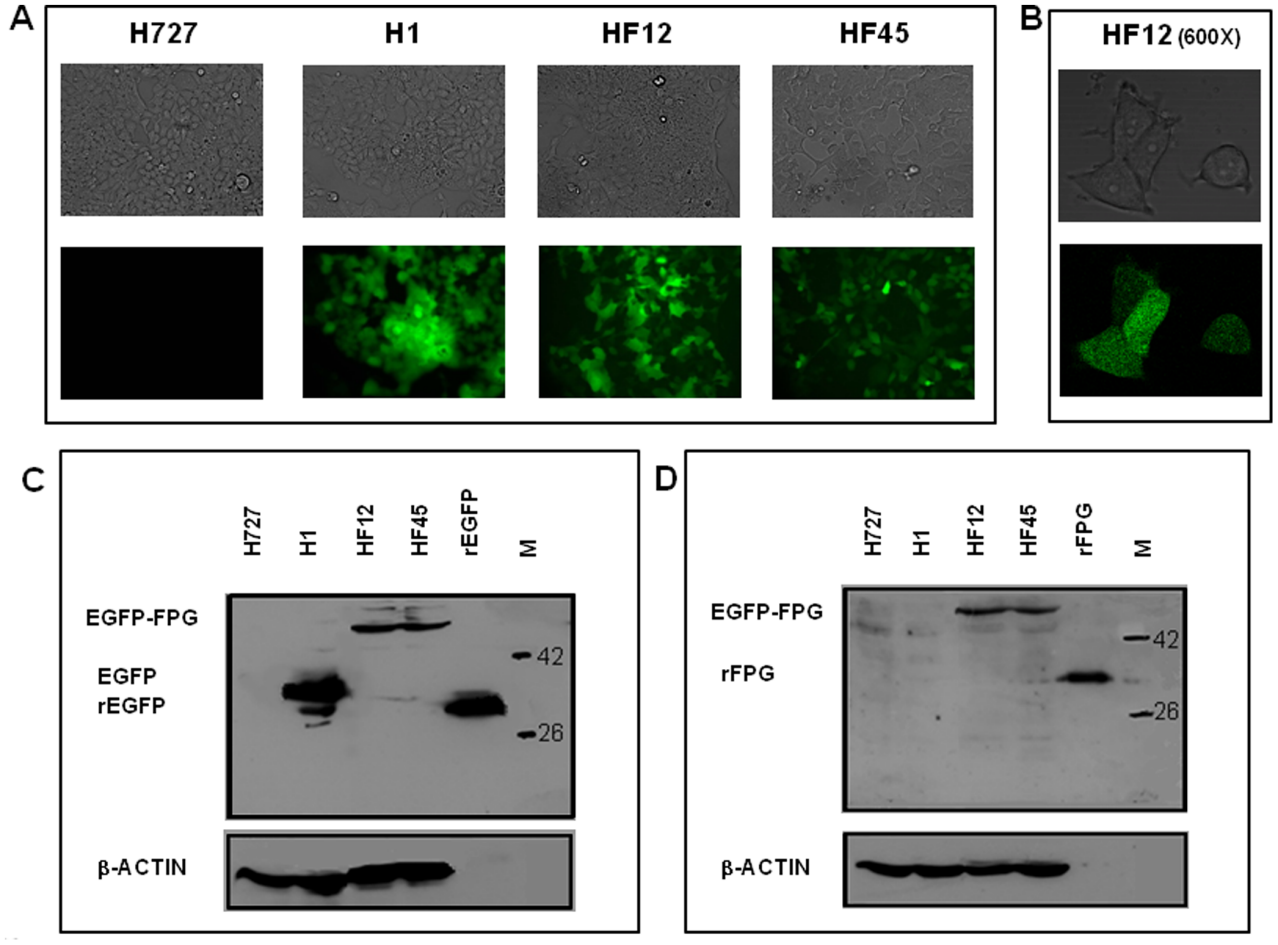
FPG expression in human bronchial carcinoid H727 cells and transfected derivatives. (A) Fluorescence analysis. Fluorescence of cells was observed under an inverted fluorescence microscope at 100X magnification and images were taken with a digital color camera. H727, untransfected cells; H1, cells transfected with the pEGFP-C1 vector; HF12, HF45, cells transfected with the pEGFP-C1-FPG vector. (B) HF12 cells analyzed by confocal microscopy at 600X magnification showing distribution of EGFP-FPG protein in both the cytoplasm and nucleus. (C) and (D) Immunodetection analyses. Experiments were performed as described under Materials and Methods. Molecular weight markers were in the far-right lane (M) and β-actin was immunodetected as a loading control. (C) Immunodetection of EGFP. Recombinant pure EGFP protein (rEGFP) was run as an internal standard. (D) Immunodetection of FPG. Recombinant pure FPG protein (rFPG) was run as an internal standard.

Figure 2 shows the expression of EGFP–FPG in HF12 and HF45 cells as determined by fluorescence microscopy (Figure 2A,B) and immunodetection (Fig. 2C,D). The original cell line H727 displayed no background fluorescence under the inverted microscope (Figure 2A) and no immunodetectable EGFP or EGFP-FPG protein (Figures 2C and 2D). The control cell line H1 transfected with the pEGFP-C1 vector and expressing the EGFP protein showed fluorescence under the inverted microscope (Figure 2A). A protein reacting with anti-EGFP antibody (M.W. 32.7 KDa – Figure 2C) but not with anti - FPG antibodies (Figure 2D) could be detected by immunodetection. The HF12 and HF45 cell lines, transfected with the pEGFP-C1-FPG vector and expressing the fusion protein EGFP–FPG showed fluorescence under the inverted microscope (Figure 2A). The fusion protein EGFP-FPG was expressed in both the cytoplasm and nuclei of HF12 and HF45 cells, as determined by confocal microscopy (Figure 2B). In these cells, a 62.9 KDa fusion protein reacting with both anti-EGFP (Figure 2C) and anti-FPG (Figure 2D) antibodies was immunodetected. In addition, 37 ng of recombinant EGFP (rEGFP - Figure 2C) or 29 ng FPG (rFPG - Figure 2D) were run in the fifth lane from left, for control purposes. The faster migration of rEGFP in comparison to the EGFP protein produced in H1 cells could be linked to post-translational modifications of the latter or a delaying effect of additional extract proteins in the loaded sample [11]. No substantial decline of EGFP–FPG expression was observed in HF12 and HF45 cells after >2 weeks of continuous culture (not shown).

### Accelerated DNA Repair

**Figure 3:**
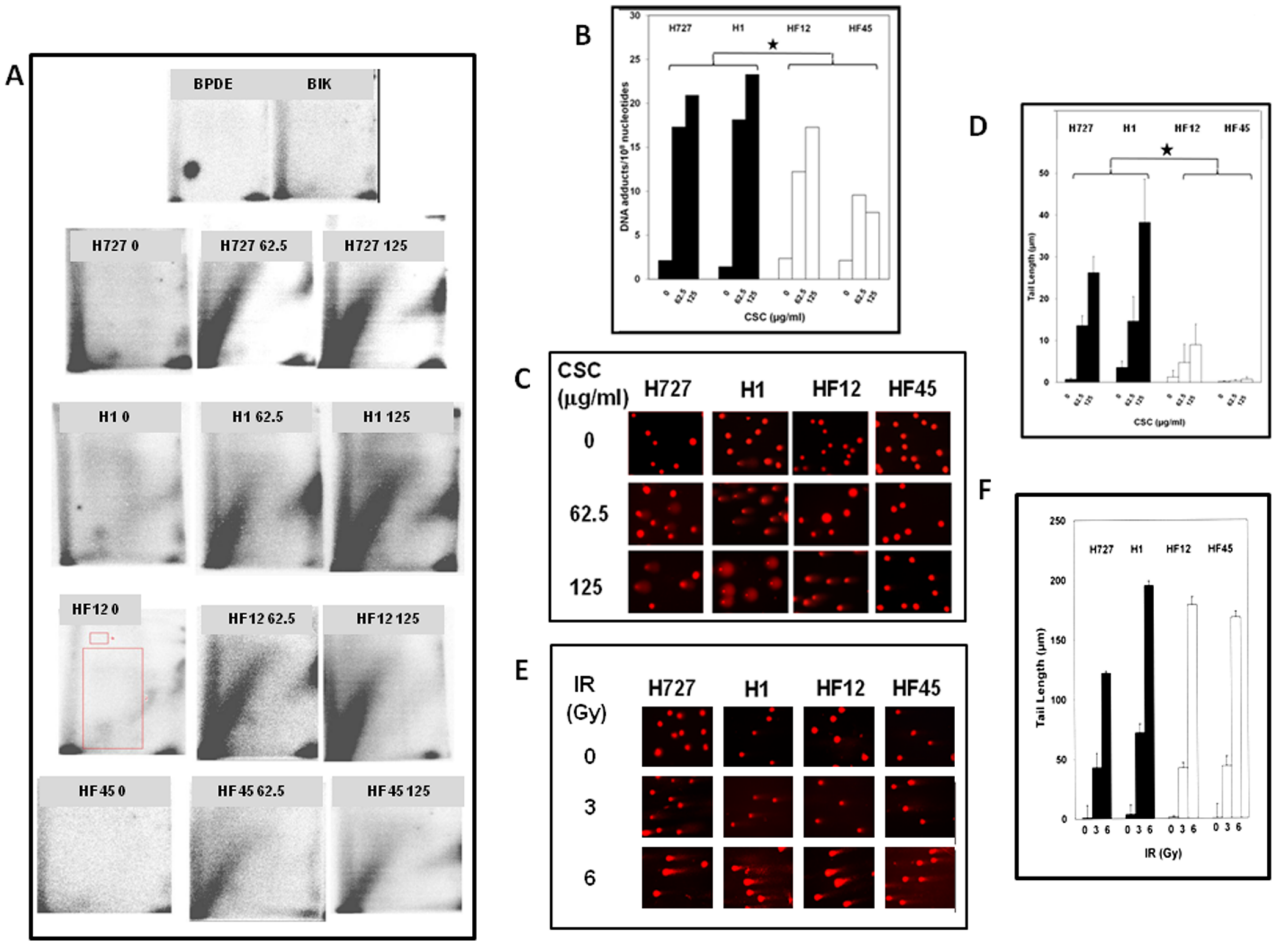
DNA repair in FPG-expressing human bronchial carcinoid H727 cells. (A) Repair of DNA adducts. Experiments were performed as described under Materials and Methods. BPDE, benzo(a)pyrene-diolepoxide-N2-deoxyguanosine adduct (positive control); Blk, DNA-free blank sample (negative control). (B) Quantification of DNA adducts. FPG transfection (pooled data of HF12 and HF45 cell lines) significantly (*, P<0.05) decreased CSC-induced DNA adducts as compared to control cell lines with no FPG (pooled data of H727 and H1). (C) Repair of CSC-induced DNA breaks. SCGE was carried out under alkaline conditions as described under Materials and Methods. (D) Quantification of CSC-induced DNA breaks. FPG transfection (pooled data of HF12 and HF45 cell lines) significantly (*, P<0.05) decreased breaks as compared to control cell lines with no FPG (pooled data of H727 and H1). (E) Induction of DNA breaks by IR. SCGE was carried out 20 minutes after treatment, as described under Materials and Methods. (F) Quantification of IR-induced DNA breaks.

Figure 3A,B summarizes the results relative to the ^32^P postlabelling analysis. Autoradiographies reported in Fig. 3A, report the typical hallmark of CSC exposure, i.e. the diagonal radioactive zone. Figure 3A panel HF12 0, highlights in red the chromatographic area used in all samples for DNA adduct quantification (great rectangle) and the area used for background subtraction (small rectangle). The diagonal radioactive zone is composed of approximately 300 DNA adducts of different composition, which cannot be separated by thin layer chromatography. As far as concerns the dose response relationship, the quantification of DNA adduct reflects a good dose–response relationship (Fig. 3B) in untransfected cell lines (H727, H1) with higher amounts of DNA adducts in cells treated with 125 µg/ml than in cells treated with 62.5 µg/ml CSC. A similar situation occurs in HF12 FPG-transfected cells. In HF45 FPG-transfected cells no significant difference was observed between DNA adducts amount as detected at the two CSC treatment doses. This absence of a strict dose-response relationship is attributable to the efficacy of the protective mechanism exerted by FPG transfection in this clone. Indeed, comparing the two FPG-transfected clones, albeit not statistically significant there was a trend towards higher efficiency of DNA adduct repair in clone HF45 as compared to clone HF12 (Figures 3A,B). FPG transfection significantly (P<0.05) decreased DNA adducts induced by CSC, as compared to control cell lines with no FPG (19.9±1.36 adducts/10^8^ normal nucleotides in pooled data from H727 and H1 cells, as compared to 11.7±2.1 adducts/10^8^ normal nucleotides in pooled data from HF12 and HF45 cells).

Figures 3C,D show the results relative to the SCGE assay after exposure of cells to CSC. We report the results as tail length (TL) being the most sensitive indicator of DNA damage in a preliminary data analysis. Likewise DNA adducts, FPG transfection significantly (P<0.05) decreased DNA breaks induced by CSC, as compared to control cell lines with no FPG (23.2±5.8 µm TL in pooled data from H727 and H1 cells, as compared to 3.7±2.0 µm TL in pooled data from HF12 and HF45 cells). Again, the HF45 line repaired DNA breaks better than HF12 (Figures 3C,D). Similar and higher levels of DNA breaks were induced after IR doses (3 and 6 Gy, respectively) that do not significantly affect the viability of H727, H1, HF12 and HF45 cells (Figures 3E,F and data not shown).

Taken together, these results indicate that expression of EGFP-FPG significantly enhances the repair of DNA damage induced by CSC in human lung cells.

### Reduced CSC Mutagenicity

**Figure 4:**
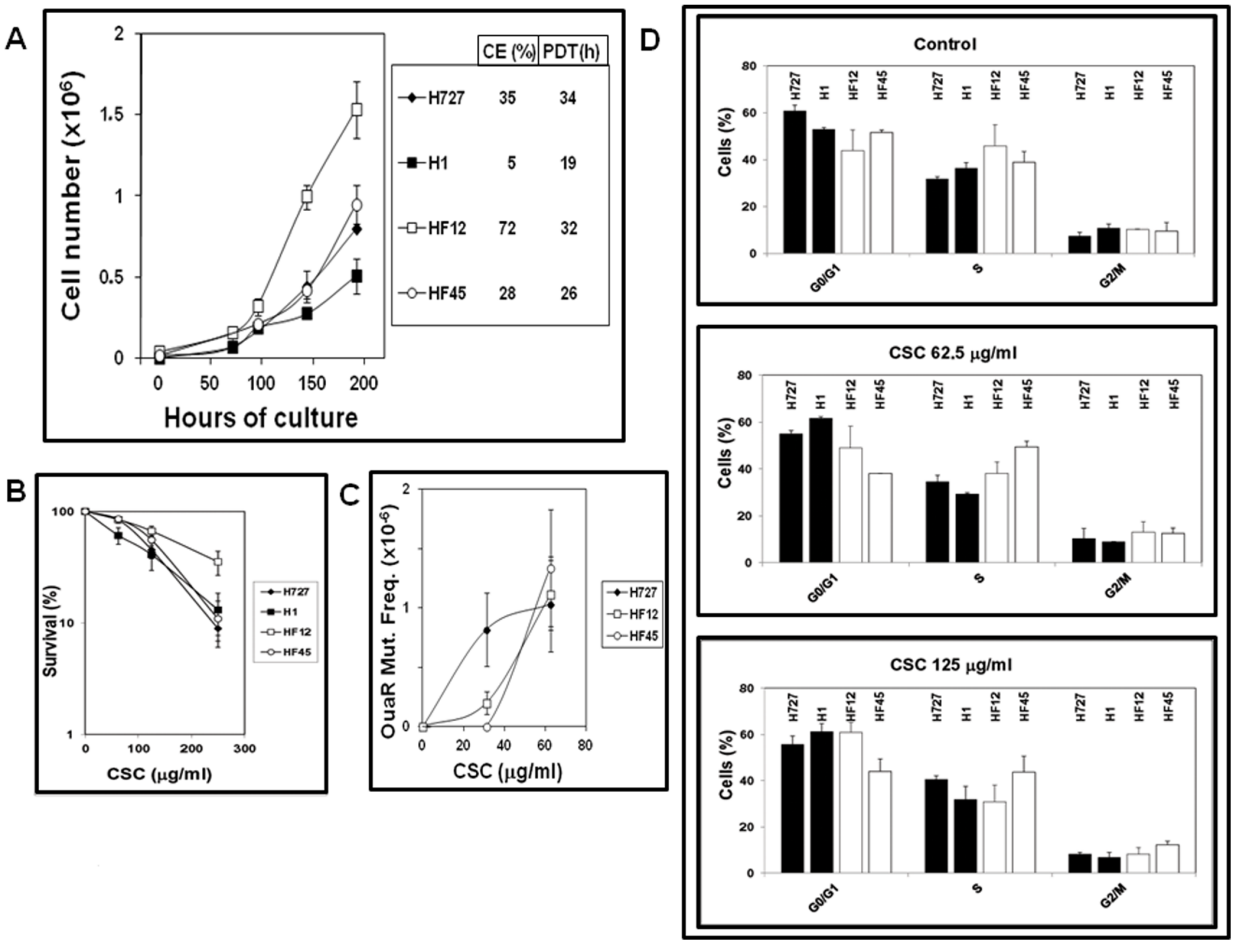
Phenotype of FPG-expressing human bronchial carcinoid H727 cells. (A) Proliferative activity. Data are the means ± SEM of three independent experiments. CE was calculated by seeding 100–500 cells and counting developed colonies three weeks later. The population doubling time (PDT) was calculated according to Glaab and Tindall [16]. (B) CSC toxicity was evaluated by the In Vitro Toxicology Assay Kit MTT-based. Data are the means ± SEM of at least three independent experiments. (C) CSC mutagenicity was determined at the *Na^+^/K^+^ ATPase* locus (oua^r^) as described under Materials and Methods. Data are the means ± SEM of at least three independent experiments. (D) Cell cycle distribution was determined by cytofluorimetry. No significant variation of cell cycle distribution was induced by CSC treatments at either 62.5 or 125 µg/ml doses.

Figure 4A shows the proliferation rate of H727 cells and derivative cell lines. Substantial differences in CE were observed among the cell lines, ranging from 5% in H1 to 72% in HF12. Less marked differences were observed in the population doubling times (PDT) which ranged from 19 (H1) to 34 (H727) hours.

In comparison to parental H727 and EGFP-expressing H1 cells [showing doses reducing survival to 37% of control (D_37_) equal to 142 and 137 µg/ml, respectively], a significant increase of resistance to cytotoxicity of CSC was observed in HF12 cells (D_37_ = 241 µg/ml), but not in HF45 cells (D_37_ = 159 µg/ml) (Figure 4B).

The protecting effect exerted by FPG from point mutagenicity induced by CSC was determined at the *Na^+^/K^+^ ATPase* locus (oua^r^ – Figure 4C). In comparison to H727 cells (solid symbols), mutant frequencies induced by the lowest used CSC concentration (31.2 µg/ml) were ablated in HF45 (open circles) and reduced 4-fold in HF12 cells (squares). No significant difference was observed at the 62.5 µg/ml concentration, indicating that the FPG-linked antimutagenic capacity could be saturated by elevated amounts of CSC-induced DNA damage. No significant differences in cell cycle distribution were observed among the cell lines under control conditions (Figure 4D, top) or after treatment with 62.5 (Figure 4D, middle) and 125 (Figure 4D, bottom) µg/ml CSC, indicating that the reductions of mutant frequencies in HF12 and HF45 with respect to H727 were not linked to variations in cell cycle distribution after CSC treatment.

## Discussion

We report that the DNA repair capacity of human lung cells may be increased by a transgenic approach utilizing *E.coli* FPG. This bacterial enzyme was chosen for possessing about 80-fold higher k(cat)/K(m) values for substrate lesions such as 7,8-dihydro-8-oxoguanine (8-oxoG) as compared to its mammalian homologue 8-oxoG DNA glycosylase (OGG1) [20]. Bacteria often repair DNA better then humans, due to their ability to cope with marked and sudden variations of intercellular environment. The results described here using FPG might not yet be parallel with results obtained with the mammalian homologue OGG1 given protein-protein interactions that might differ between the *E. coli* and mammalian proteins.

FPG uses its N-terminal proline (Pro2) as a nucleophile in the glycosylase/AP-lyase reaction [12] [13]. In the plasmid construct used in this and previous studies, proline 2 is saved (Figure 1) and its nucleophilic function is not significantly affected by the N-terminal fusion of the EGFP tag as demonstrated by the enhanced DNA repair of 8-oxodG, 5-OHdC and AP sites after expression of EGFP-FPG in different human cells measured by a number of DNA repair assays [11], [14], [21], [22]. Consistently, the mutation frequency induced by oxidizing agents at the *Na^+^-K^+^ ATPase* locus in human cells expressing EGFP-FPG is reduced by about one order of magnitude in comparison to cells transfected with vector only [11].

In agreement with previous observations [11], the frequency of clones in the H727 adenocarcinoma cell line displaying detectable expression of the fusion protein EGFP–FPG was relatively low. Only nineteen stably fluorescent clones were isolated after transfection of ∼200,000 H727 cells, and the HF12 and HF45 clones were selected for further characterization due to their higher expression of EGFP-FPG protein (as determined by fluorescence analysis and immunodetection) and proliferative capacity (as determined by PDT), the latter being higher in HF45 than in HF12 (Figures 2 and 4). Elevated expression of EGFP-FPG and proliferative capacity may thus seldom occur concomitantly, when protein expression is achieved by the pEGFP-C1 DNA plasmid vector, due to a number of reasons including transcriptional silencing of the gene, elimination of the gene from the host genome and negative selection of cells overexpressing the fusion protein [11], [14], [23]. Regardless of limits imposed by the type of vector employed, several studies have demonstrated yet that the expression of FPG is possible in multiple types of mammalian cells and tissues with protection from the cytotoxic and mutagenic effects of a wide range of DNA-damaging agents [11], [14], [21], [22], [24]–[27]. The fusion protein EGFP-FPG was expressed in both the cytoplasm and nuclei of HF12 and HF45 cells, in agreement with the known capacity of EGFP to translocate into the nucleus (Figure 2B [28]). In agreement with previous studies, the EGFP-FPG protein expression was stable for at least 15 days of continuous cell culture, indicating that expression of this fusion protein is relatively well tolerated by human cells [11], [14], [21].

In view of the broad substrate specificity and elevated kinetics parameters of FPG protein in comparison to human 7,8-dihydro-8-oxoguanine DNA glycosylase (hOGG1) [20], it was anticipated that FPG-expressing cells could attain improved DNA repair capacity of CSC-induced lesions, although our results do not allow to identify them individually (Figure 3). There are over 70 carcinogens in CSC, belonging to different chemical classes such as polycyclic aromatic hydrocarbons (PAH) and their heterocyclic analogues, aromatic amines and N-nitrosamines [2]. Most of those compounds produce DNA adducts of different size, toxic and mutagenic power. FPG possesses a versatile catalytic site allowing accommodation and removal of a number of different adducts [5]. The glycolysis of CS-induced adducts by FPG occurs with varying efficacy: some oxidized and small adducted purines (e.g. 8-oxodG and N7-Me-FapydG) are excised from the DNA backbone faster than some oxidized pyrimidines (e.g. DHU and 5OHdC) that in turn are excised faster than some bulky adducts such as the imidazole ring-opened derivative of N-(deoxyguanosine-8-yl)-2-aminofluorene (C8-AF-irodG) or the ring-opened N-7 guanine adduct produced by sulfur mustard, till the unproductive mode by which FPG recognizes and binds N7-Benzyl-FapydG [5], [6], [8], [9]. In this regard, the rate of imidazole ring-opening of purines occurs easily when adducts bind the N7 position of guanine, even at neutral pH, suggesting that a significant fraction of CSC-induced FPG substrates may be represented by ring-opened purines [29]. For instance, withdrawal of electrons from the purine ring by an alkyl group destabilizes the imidazole ring with formation of 2,6-diamino-4-hydroxy-5N-alkyl-formamidopyrimidine (Fapy-7AlkG) [30]. The ^32^P post-labeling assay with butanol enrichment used in our study allows the detection of adducts from different CS components including PAH, aromatic amines, heterocyclic amines, alkenylbenzene derivatives, benzene and its metabolites, styrene, simple alkylating agents and reactive oxygen species [31]. In particular, butanol enrichment preferentially extracts bulky lipophilic adducts not including Fapy lesions or 8-oxodG but including lipid peroxidative derivatives among which the ring-opened derivative of ethenoadenine has been demonstrated to be recognized by FPG [32], [33]. The oxidative nature of the diagonal radioactive zone occurring in cells exposed to CSC is established: antioxidants such as N-acetylcysteine are quite effective in preventing diagonal radioactive zone formation in CS-exposed organisms [34].

The data presented here show that FPG expression may significantly reduce the total amount of CS-induced adducts (Figure 3A,B) in a productive mode up to completion of the DNA sealing step, as demonstrated by the significant reduction of CS-induced DNA breaks in FPG expressing clones (Figures 3C,D). Similar or higher levels of DNA breaks can be induced by IR doses that do not significantly affect viability of H727 cells, where constitutive activation of the phosphatidylinositol 3 kinase-Akt pathway, a known mechanism of radiation resistance, has been demonstrated (Figures 3E,F) [35], [36]. Taken together, our data indicate that FPG expression may potentiate the cell’s capacity to counteract the persistence of mutagenic (but not of toxic) CS-induced DNA adducts. The identification of individual types of repaired lesions (probably an elevated number) is beyond the aim of the present work and will require future studies. Development of appropriate vectors for expression of FPG in bronchioalveolar stem cells of individuals at risk for CS.induced-lung cancer, may confer a possible translational significance to our results [37].

A position effect of vector integration might be called into question to explain the higher resistance of HF12 to CSC toxicity as compared to HF45, despite its lower DNA repair capacity (Figure 4B). Mechanisms involved in resistance to CSC toxicity other than DNA repair [e.g. linked to the elevated CE observed in HF12 (Figure 4A)] may have been enhanced by vector integration close to genes involved in such mechanisms. Further studies are certainly required to deepen our understanding of unidentified factors influencing resistance to CS which may further affect adducts formation and their release. Being common to both HF12 and HF45 clones and in line with previous observations on human bladder carcinoma cells [11], the increased DNA repair capacity for CS-induced damage is the most likely explanation for the lower mutant frequency induced by a low concentration (31.2 µg/ml) of CS at the *Na^+^/K^+^ ATPase* locus (oua^r^) in FPG-expressing clones as compared to parental H727. No mutant frequency could be measured in EGFP-expressing H1 cells, due to their very low capacity to form colonies (CE = 5% - Figure 4A) that in turn could be ascribed, at least in part, to the elevated expression of the foreign tag EGFP. Overexpression of some BER components (e.g. N-methylpurine-DNA glycosylase or DNA polymerase beta), has been shown to imbalance the pathway and lead to more unrepaired or mis-repaired DNA damage [38] [39]. This is not the case with FPG: the antimutagenic role of expression of bacterial FPG in mammalian cells has been repeatedly confirmed (reviewed in [10]). Merely to quote a few examples, FPG expression reduces gamma rays-induced mutations at the *HPRT* locus of mammalian cells and affords protection from the mutagenic effects of various chemotherapeutic regimens [24] ^,^ [27] ^,^ [40]. The reduced oua^r^ mutant frequency observed in HF12 and HF45 cells is in agreement with those previous findings and extends the protective role of FPG to CS-induced mutations. No difference of oua^r^ mutant frequency was observed between EGFP-FPG expressing clones and parental cells at the higher 62.5 µg/ml. At this CS concentration, the repair of CS-induced bulky adducts and DNA breaks was not saturated yet (Figure 3). This discrepancy between the CS concentration values saturating the protection from CS mutagenicity on one side and the FPG-enhanced DNA repair on the other side may indicate that only a part of mutations induced by CS are due to the lesions recognized by FPG.

## Conclusion

Taken together, the results of this study indicate that FPG expression accelerates the repair of a fraction of CS – induced DNA adducts in human lung cells conferring partial permanent protection from CS mutagenicity.
